# β2 nAChR Activation on VTA DA Neurons Is Sufficient for Nicotine Reinforcement in Rats

**DOI:** 10.1523/ENEURO.0449-22.2023

**Published:** 2023-05-24

**Authors:** Noah B. Walker, Yijin Yan, Melissa A. Tapia, Brenton R. Tucker, Leanne N. Thomas, Brianna E. George, Alyssa M. West, Christopher B. Marotta, Henry A. Lester, Dennis A. Dougherty, Katherine M. Holleran, Sara R. Jones, Ryan M. Drenan

**Affiliations:** 1 Department of Physiology and Pharmacology, Wake Forest University School of Medicine, Winston-Salem, NC 27157; 2 Division of Chemistry and Chemical Engineering, California Institute of Technology, Pasadena, CA 91106; 3 Division of Biology and Biological Engineering, California Institute of Technology, Pasadena, CA 91106

**Keywords:** acetylcholine, addiction, dopamine, nicotine, reinforcement, tobacco

## Abstract

Mesolimbic nicotinic acetylcholine receptor (nAChRs) activation is necessary for nicotine reinforcement behavior, but it is unknown whether selective activation of nAChRs in the dopamine (DA) reward pathway is sufficient to support nicotine reinforcement. In this study, we tested the hypothesis that activation of β2-containing (β2*) nAChRs on VTA neurons is sufficient for intravenous nicotine self-administration (SA). We expressed β2 nAChR subunits with enhanced sensitivity to nicotine (referred to as β2Leu9′Ser) in the VTA of male Sprague Dawley (SD) rats, enabling very low concentrations of nicotine to selectively activate β2* nAChRs on transduced neurons. Rats expressing β2Leu9′Ser subunits acquired nicotine SA at 1.5 μg/kg/infusion, a dose too low to support acquisition in control rats. Saline substitution extinguished responding for 1.5 μg/kg/inf, verifying that this dose was reinforcing. β2Leu9′Ser nAChRs also supported acquisition at the typical training dose in rats (30 μg/kg/inf) and reducing the dose to 1.5 μg/kg/inf caused a significant increase in the rate of nicotine SA. Viral expression of β2Leu9′Ser subunits only in VTA DA neurons (via TH-Cre rats) also enabled acquisition of nicotine SA at 1.5 μg/kg/inf, and saline substitution significantly attenuated responding. Next, we examined electrically-evoked DA release in slices from β2Leu9′Ser rats with a history of nicotine SA. Single-pulse evoked DA release and DA uptake rate were reduced in β2Leu9′Ser NAc slices, but relative increases in DA following a train of stimuli were preserved. These results are the first to report that β2* nAChR activation on VTA neurons is sufficient for nicotine reinforcement in rats.

## Significance Statement

Nicotinic acetylcholine receptor (nAChR) pharmacology and neurobiology in the dopamine (DA) reward pathway is complex and it has been a challenge to identify the minimum receptor/circuit combination(s) giving rise to nicotine dependence. This study reveals that activation of β2-containing nAChRs in ventral tegmental area dopamine neurons is sufficient to support acquisition and maintenance of nicotine self-administration (SA) in rats. This work, which employs a gain-of-function approach, complements and extends prior loss-of-function experiments demonstrating the importance of these receptors in several nicotine-related behaviors. This study (1) affirms the importance of β2 nAChRs in nicotine reinforcement, and (2) provides a useful *in vivo* approach for developing nicotine dependence therapeutics with either nicotinic or non-nicotinic mechanisms of action.

## Introduction

Nicotine, the key psychoactive compound in tobacco products, is paradoxical; it has potent psychostimulant properties but is nevertheless a weak reinforcer. A variety of behavioral, pharmacological, genetic, and circuit-based approaches have been used to examine this issue with the goal of understanding the mechanistic basis for nicotine dependence/addiction. Identification of nicotinic acetylcholine receptor (nAChR) subtypes responsible for nicotine’s reinforcing and dependence-producing property has been of great interest, as accomplishing this task may lead to new drugs for improved smoking cessation outcomes. Most current knowledge comes from loss-of-function experiments. For example, Corrigall used a pharmacological approach to demonstrate that β2* nAChR activity is necessary for nicotine self-administration (SA) in a rat model (Corrigall et al., 1994). Using knock-out (KO) mice, Picciotto and colleagues identified the β2 nAChR subunit as necessary for the transfer of operant responding for cocaine to responding for nicotine (Picciotto et al., 1998). Using an active versus yoked paradigm with mice having only a single nicotine IVSA session, Changeux and colleagues identified the β2 nAChR subunit as necessary for nicotine self-administration (Pons et al., 2008). These loss-of-function studies indicate the importance of β2 nAChR subunits in nicotine reward and self-administration behavior, but they do not address the question of β2 sufficiency.

nAChR gain-of-function approaches, involving expression of nAChR subunits with increased sensitivity to agonist, complement loss-of-function experiments and provide a useful and alternative method to examine the molecular basis for nicotine-related behaviors (Drenan and Lester, 2012). Early molecular pharmacology work on nAChRs that focused on understanding the gating mechanism employed mutagenesis of a nearly universally conserved leucine residue (the 9′ Leucine) in the TM2 α-helix, resulting in a slowing/reduction in desensitization and an increase in sensitivity (Revah et al., 1991; Bertrand et al., 1992; Yakel et al., 1993; Filatov and White, 1995). This effect is thought to arise from stabilization of the open state, and a serine substitution (used in this study) strongly induces the effect whereas alanine or hydrophobic substitutions are more modest (Revah et al., 1991; Bertrand et al., 1992; Yakel et al., 1993).

A key discovery related to the present work is that Leu9′ mutant subunits incorporate into pentameric receptors with either wild-type (WT) or other Leu9′ mutant subunits, and receptor sensitivity (or hypersensitivity) is directly related to the number of Leu9′ mutant subunits in each pentamer (Labarca et al., 1995). Leu9′ mutant subunits can therefore be expressed on a background of WT subunits, making them useful for *in vivo* experiments in behaving animals.

nAChR subunits modified at the Leu9′ position have been useful for understanding nicotine-related behaviors and neurobiology. Conditioned place preference experiments with knock-in mice expressing Leu9′Ala α4 subunits demonstrated that activation of α4β2 nAChRs is sufficient for nicotine reward-like behavior (Tapper et al., 2004). Studies with transgenic mice expressing Leu9′Ser α6 subunits revealed a myriad of behavioral/pharmacological functions for α6β2 nAChRs (Drenan et al., 2008, 2010; Cohen et al., 2012; Engle et al., 2013, 2015; Powers et al., 2013; Wang et al., 2014; Berry et al., 2015; Wieskopf et al., 2015). However, nicotine self-administration is difficult to establish in mice, so the Leu9′ approach has not been fully used to make key insights into pharmacological/cellular/circuit mechanisms of nicotine reinforcement.

To address this gap in the field, we employed the Leu9′ approach to answer the following questions: is selective activation of VTA β2 nAChRs sufficient for acquisition of nicotine self-administration and if so, is β2 nAChR activation reinforcing? We designed and produced an adeno-associated virus (AAV) for expression of rat β2 nAChR subunits with a Leu9′ to Serine substitution. This vector was delivered via stereotaxic surgery to the brain of Sprague Dawley (SD) or transgenic TH-Cre Long–Evans rats, triggering expression of hypersensitive β2 nAChRs for subsequent intravenous nicotine self-administration experiments and physiology studies.

## Materials and Methods

### Materials

Nicotine hydrogen tartrate salt was obtained from Glentham Life Sciences (catalog #GL9693-5G). Injectable heparin sodium (catalog #07-892-8971) and injectable meloxicam (catalog #07-891-7959) were obtained from Patterson Veterinary Supply. Acetylcholine (ACh) chloride and atropine sulfate (atropine) were purchased from Millipore Sigma. QX314 chloride (QX314) was obtained from Tocris Bioscience.

### Molecular biology

We conceived of and designed the AAV5.2-hSyn-DIO-Chrnb2Leu273Ser-P2A-GFP (β2 Leu9′Ser) vector, which was then prepared by Virovek AAV5-hSyn-mCherry-Cre was obtained from the University of North Carolina Vector Core Facility. Rat α4 and β2 nAChR subunits were cloned into the pGEMhe vector (Marotta et al., 2014). The Leu9′Ser mutation was introduced into the β2 subunit vector via the QuikChange protocol (Stratagene). The α4 and β2 circular DNA vectors were linearized using the Sda I (Sbf I) enzyme (Thermo Fischer Scientific). Qiaquick PCR Purification kit (QIAGEN) was used to purify the linearized DNA. mRNA was transcribed *in vitro* from the linearized DNA using the mMessage Machine T7 Transcription kit (Ambion). The resultant mRNA was isolated using the RNeasy RNA Purification kit (QIAGEN).

### Animals

All experimental protocols involving vertebrates were reviewed and approved by the Wake Forest University School of Medicine Institutional Animal Care and Use Committee. Procedures also followed the guidelines for the care and use of animals provided by the National Institutes of Health Office of Laboratory Animal Welfare. All efforts were made to minimize animal distress and suffering during experimental procedures, including during the use of anesthesia. A total of *n* = 85 male SD rats (Envigo) were used. SD rats were ∼300 g (approximately eight weeks old) when they arrived at our facility. Rats were housed at 22°C on a reverse 12/12 h light/dark cycle (4 P.M. lights on, 4 A.M. lights off). Transgenic Long–Evans TH::Cre rats (Witten et al., 2010, 2011) were obtained from Rat Resource and Research Center (RRRC). A breeding colony was established for TH-Cre rats by breeding heterozygous TH-Cre males with WT Long–Evans females (obtained from Envigo). All progeny from breeding pairs were genotyped (Transnetyx), and nontransgenic littermates were used as controls. A total of *n* = 20 male TH-Cre (*n* = 14) or non-Tg littermate (*n* = 6) rats were used for experiments. *Xenopus laevis* oocytes were purchased from EcoCyte Bioscience.

### Apparati

Rats were trained in Med Associates operant chambers (interior dimensions, in inches: 11.9 × 9.4 × 11.3, catalog #MED-007-CT-B1) located within sound-attenuating cabinets. The SA system was housed in a dedicated room within the same laboratory suite as the rat’s housing room. A PC computer was used to control the SA system via Med PC IV software. Each chamber had transparent plastic walls, a stainless-steel grid floor, and was equipped on the right-side wall with two nose pokes (2.4 inches from grid floor to nose poke center) which flanked a pellet receptacle coupled to a pellet dispenser. A white stimulus light was located above each nose poke, and a house light was located at the top of the chamber on the left-side wall. During food and drug SA sessions, nose pokes on the active nose poke activated either the pellet dispenser or an infusion pump, respectively. Nose pokes on the inactive nose poke had no consequence. For intravenous drug infusions, each rat’s catheter was connected to a liquid swivel via polyethylene tubing protected by a metal spring. The liquid swivel was connected to a 10-ml syringe loaded onto the syringe pump.

### Operant food training

Approximately one week after arrival, pair-housed rats were food-restricted for several days to enhance their participation in operant responding. Rats were fed standard chow (LabDiet Prolab RMH 3000 5P00, catalog #0001495; 40 g per cage) once per day at least 1 h after finishing testing. Water was available *ad libitum* except during operant behavioral sessions. Food training sessions were 1 h in duration, and rats were trained to nose poke for food pellets (45 mg; Bio-Serv Dustless Precision Pellets, catalog #F0021) on the same nose poke that would subsequently be paired with drug infusions in nicotine IVSA sessions. A fixed ratio 1 (FR1; no timeout) schedule was used for food training; no visual cues (stimulus light, house light) were illuminated during the session and rats could earn a maximum of 75 food pellets during the 1-h session. Once each rat successfully earned at least 50 pellets with at least a 2:1 preference for the active nose poke over the inactive nose poke, no further food training was conducted. Rats met this criterion on average between 3 and 5 d. Food training was used to shorten the time required for rats to acquire nicotine SA, though it is not required.

### Stereotaxic surgery

After arrival at our facility, rats were anesthetized with isoflurane (3% induction, 2–5% maintenance) and were introduced into a stereotaxic rat frame. Rats received a bilateral injection into the VTA using coordinates from bregma (SD rats: AP: −5.25, ML: ±1.00, DV: −8.00; TH::Cre rats: AP: −5.40, ML: ±0.70, DV: −8.20). Coordinates were derived from “Brain Maps 4.0” (Larry Swanson; University of Southern California; Swanson, 2018). Virus was infused using a 22-gauge Hamilton injection syringe at a rate of 100 nl/min and the injection needle was left in place for 5 min after each injection before gradually being retracted. Rats were then given 6–7 d for recovery.

### Indwelling jugular catheter surgery

After acquiring food operant responding, rats were anesthetized with isoflurane (3% induction, 2–3% maintenance) and implanted with indwelling jugular catheters (Instech, catalog #C30PU-RJV1402). Meloxicam (2 mg/kg) was administered postoperatively to relieve pain and reduce inflammation. Rats were singly housed following surgery and throughout all SA procedures. Rats were allowed 7 d for recovery from surgery, and catheters were flushed several times during this recovery period with heparin sodium dissolved in sterile saline.

### Intravenous drug self-administration

After recovery from catheter surgery, rats were allowed to self-administer saline or nicotine (dose variable, as described in Results) in a volume of 0.035 ml over 2 s during 2-h SA sessions, Monday through Friday (no SA sessions occurred on weekends). (−)-Nicotine hydrogen tartrate salt (Glentham Life Sciences) was dissolved in sterile saline, and the pH was adjusted to 7.4. Infusions, delivered by an infusion pump, were triggered by one nose poke response on the active nose poke. Infusions (2-s duration) were simultaneously paired with illumination of the stimulus light over the active nose poke for 3 s. An active nose poke response that resulted in an infusion extinguished the house light for a 20-s timeout period (TO-20s), during which responding was recorded but had no consequence. Responses on the inactive nose poke were recorded but had no scheduled consequence. At the end of the session, the house light was extinguished and responding had no consequences. Rats were removed from the training chambers as soon as possible after the end of the 2-h session and were rationed to 20 g of standard lab chow at least 1 h after finishing their sessions. Modified chow availability was used throughout SA and a range of 85–90% of free-feeding body weight was maintained. SD rats were allowed to self-administer nicotine (30 μg/kg/inf or 1.5 μg/kg/inf) for 10 d on an FR1/TO-20s schedule of reinforcement. For rats receiving 30 μg/kg/inf, *n* = 2 of 8 injected SD β2Leu9′Ser::Cre(−) rats, *n* = 2 of 10 injected SD β2Leu9′Ser::Cre(+) rat, and *n* = 0 of 7 control SD rats failed acquisition. Acquisition failure could be triggered by the occurrence of any one of the following: (1) <10 nicotine infusions were earned within the 2-h session for two or more consecutive sessions, (2) the ratio of active to inactive nose pokes was <2.0 for three or more consecutive sessions, and (3) a drop of 75% or greater in responding on the active nose poke occurred during sessions #6–10 of acquisition. For SD β2Leu9′Ser::Cre(+) rats receiving 1.5 μg/kg/inf nicotine, *n* = 8 of 36 injected rats failed acquisition because of a failure to maintain an average of 11.1 infusions across SA days 6–10. This value is based on our prior work with saline SA, where naive SD rats typically average this many saline self-infusions across SA days 6–10 under the identical operant/cue conditions. Using this same metric, *n* = 7 of 8 injected SD β2Leu9′Ser::Cre(−) rats receiving 1.5 μg/kg/inf nicotine failed to acquire SA, and *n* = 9 (of nine total) SD control rats receiving 1.5 μg/kg/inf nicotine failed to acquire SA. For Long–Evans TH-Cre rat nicotine (at 1.5 μg/kg/inf) SA acquisition, we calculated the average # of infusions across SA days 11–17 in *n* = 6 non-Tg littermate rats injected with β2Leu9′Ser vectors. This value, plus 2× the SD (equal to approximately six infusions), was used as a cutoff to determine acquisition in *n* = 11 TH-Cre rats injected with β2Leu9′Ser vectors. Based on this criterion, *n* = 1 of 11 total injected TH-Cre rats failed acquisition because of an average # of infusions across days 11–17 below 6.

### Immunohistochemistry

Rats were deeply anesthetized with isoflurane and perfused transcardially with 50 ml of PBS followed by 250 ml of 4% paraformaldehyde (PFA) in PBS. Brains were removed and stored at 4°C overnight in PBS containing 4% PFA and 4% sucrose (to dehydrate tissue). Coronal sections (30 μm) were cut in triplicate on a microtome and collected into 12-well tissue culture plates filled with PBS and 0.1% sodium azide. Slices were washed in PBS with 0.3% Triton X-100, blocked in PBS with 0.1% Triton X-100 and 5% donkey serum for 1 h at 4°C, and incubated overnight at 4°C in PBS, 0.1% triton, and 5% donkey serum with primary antibodies. Primary antibodies (and dilutions) were as follows: 1:1000 for rabbit-anti-GFP (A11122, Invitrogen), 1:500 for rabbit-anti-dsRed (632496, Takara Bio), 1:1000 for sheep-anti-tyrosine hydroxylase (TH; AB152, EMD Millipore). Slices were then washed three times for 10 min in a mix of PBS and 0.3% Triton X-100 before a 1-h incubation in PBS, 0.1% Triton X-100, and 5% donkey serum with secondary antibodies, and three subsequent washes. Secondary antibodies (and dilutions) were as follows: 1:500 for chicken-anti rabbit Alexa Fluor 488 (A21441, Invitrogen), 1:500 for donkey-anti-rabbit Alexa Fluor 647 (A32795, Invitrogen), 1:500 for donkey-anti-sheep Alexa Fluor 647 (A21448). Sections were immediately mounted and coverslipped with sodium bicarbonate then imaged on a Nikon A1 confocal microscope.

### *Ex vivo* fast scan cyclic voltammetry

On the morning of recordings, rats were deeply anesthetized with isoflurane and decapitated. The brain was removed and immersed in oxygenated artificial CSF (aCSF) containing (in mm): 126 NaCl, 2.5 KCl, 1.2 NaH_2_PO_4_, 2.4 CaCl_2_, 1.2 MgCl_2_, 25 NaHCO_3_, 11 glucose, 0.4 l-ascorbic acid. A vibrating tissue slicer (Leica VT1200S, Leica Biosystems, Wetzler, Germany) was used to prepare 400 μm thick coronal brain slices containing the NAc core (NAcc). Slices were transferred to a recording chamber and submerged in a bath of oxygenated aCSF (32°C) perfused at a rate of 1 ml/min. A carbon-fiber microelectrode (CFE; 150–200 μm length, 7-μm radius) and bipolar stimulating electrode were placed in the NAc core. Endogenous dopamine (DA) release was electrically evoked by a single pulse (350 μA, 7.5 V, 4 ms, monophasic) applied to the slice at 5 min intervals. Extracellular dopamine was measured by applying a triangular waveform (−0.4 to + 1.2 to −0.4 V vs Ag/AgCl, 400 V/s) to the CFE. Baseline dopamine release and uptake kinetics were considered stable after an hour of slice acclimation and when the dopamine signal was stable for at least three successive collections. The effects of two different stimulation patterns on dopamine release were tested. A stimulation intensity relation was collected by using a single pulse stimulation at varying intensities, 0.5, 1.0, 1.5, 2.0, 2.5, 3.0, 3.5, 4.0, 4.5, 5.0, 6.0, 7.0, 8.0, 9.0, 10.0 V with an interstimulus interval of 60 s. A frequency-response relation was obtained using four-pulse stimulations at 3, 10, 30, and 100 Hz with 5-min interstimulus intervals. Dopamine response was determined following a concentration response curve using bath application of nicotine (1 to 100 nm), with doses increasing on a half-log scale. Once a stable dopamine signal was collected at the baseline and each dose, a single four-pulse, 30-Hz stimulation was applied for a single file collection. Demon Voltammetry and Analysis software (freely available from Wake Forest University School of Medicine) was used to analyze all FSCV data (Yorgason et al., 2011). Calibration factors for individual recording electrodes were determined using a multiple linear regression model of preestablished background currents and resulting dopamine sensitivity. The calibration factor of each electrode was used to convert the electrical current measured during experiments to dopamine concentration. Michaelis–Menten modeling was used to determine the concentration of dopamine released and the maximal rate of uptake (V_max_) following electrical stimulation.

### Two-electrode voltage clamp electrophysiology

ND96 Ca^2+^ free buffer (96 mm NaCl, 2 mm KCl, 1 mm MgCl2, 5 mm HEPES at pH 7.5) was used to generate a 1 m ACh stock solution. A series of ∼3-fold concentration steps were made and serially diluted for several orders of magnitude, totaling 12–15 doses for electrophysiology studies. A 1:2 mass ratio of α4:β2 mRNA were mixed to deliver 10 ng of total mRNA in a 50 nl injection volume. Oocytes were injected and then incubated at 18°C in ND96+ medium with 5% horse serum for 24 h. All electrophysiology recordings were performed in two-electrode voltage clamp mode on an OpusXpress 6000A (Molecular Devices). The running buffer was ND96 Ca^2+^ free and holding potentials were –60 mV for all experiments. 1 ml ACh drug applications were applied over 15 s followed by 3 min of buffer (3 ml/min). Doses were applied from lowest to highest concentrations. Data were sampled at 50 Hz and then low-passed filtered at 5 Hz. Maximum current values were recorded for ACh doses. These were normalized, averaged, and fit to a one Hill equation model to calculate EC_50_ and Hill coefficient values. Error bars represent the SEM.

### Brain slice preparation and recording solutions

Rats (*n* = 4 SD, *n* = 3 β2Leu9′Ser::Cre(+)) were anesthetized with isoflurane before trans-cardiac perfusion with oxygenated (95% O_2_/5% CO_2_), 4°C *N*-methyl-D-glucamine (NMDG)-based recovery solution that contains (in mm): 93 NMDG, 2.5 KCl, 1.2 NaH_2_PO_4_, 30 NaHCO_3_, 20 HEPES, 25 glucose, 5 sodium ascorbate, 2 thiourea, 3 sodium pyruvate, 10 MgSO_4_·7H_2_O, and 0.5 CaCl_2_·2H_2_O; 300–310 mOsm; pH 7.3–7.4. Brains were immediately dissected after the perfusion and held in oxygenated, 4°C recovery solution for 1 min before cutting a brain block containing the VTA and sectioning the brain with a vibratome (VT1200S; Leica). Coronal slices (250 μm) were sectioned through the VTA and transferred to oxygenated, 33°C recovery solution for 12 min. Slices were then kept in holding solution containing (in mm): 92 NaCl, 2.5 KCl, 1.2 NaH_2_PO_4_, 30 NaHCO_3_, 20 HEPES, 25 glucose, 5 sodium ascorbate, 2 thiourea, 3 sodium pyruvate, 2 MgSO_4_·7H_2_O, and 2 CaCl_2_·2H_2_O; 300–310 mOsm; pH 7.3–7.4 for 60 min or more before recordings. Brain slices were transferred to a recording chamber (1 ml volume), being continuously superfused at a rate of 1.5–2.0 ml/min with oxygenated 32°C recording solution. For our recording chamber and solution flow rate, we estimate that complete solution exchange occurs in 5–8 min. The recording solution contained (in mm): 124 NaCl, 2.5 KCl, 1.2 NaH_2_PO_4_, 24 NaHCO_3_, 12.5 glucose, 2 MgSO_4_·7H_2_O, 2 CaCl_2_·2H_2_O; 300–310 mOsm; pH 7.3–7.4). For puffer experiments, the recording solution was supplemented with 1 μm atropine. Patch pipettes were pulled from borosilicate glass capillary tubes (1B150F-4; World Precision Instruments) using a programmable microelectrode puller (P-97; Sutter Instrument). Tip resistance ranged from 4.0–7.0 MΩ when filled with internal solution. A potassium gluconate-based internal solution was used for recordings (in mm): 135 potassium gluconate, 5 EGTA, 0.5 CaCl_2_, 2 MgCl_2_, 10 HEPES, 2 MgATP, and 0.1 GTP; pH adjusted to 7.25 with Tris base; osmolarity adjusted to 290 mOsm with sucrose. The internal solution contained QX314 (2 mm) for improved voltage control.

### Patch clamp electrophysiology

Electrophysiology experiments were conducted using a Nikon Eclipse FN-1 upright microscope equipped with a 40× (0.8 NA) water-dipping (3.3-mm working distance) objective. Neurons in the VTA were targeted for recording. Neurons within brain slices were first visualized with infrared or visible differential interference contrast (DIC) optics. A computer running pCLAMP 10 software was used to acquire whole-cell recordings along with a Multiclamp 700B amplifier and a Digidata 1550A A/D converter (all from Molecular Devices Inc.). Data were sampled at 10 kHz and low pass filtered at 1 kHz. Immediately before giga seal formation, the junction potential between the patch pipette and the superfusion medium was nulled. Series resistance was uncompensated. To record physiological events following local application of drugs, a drug-filled pipette was moved to within 20–40 μm of the recorded neuron using a second micromanipulator. A Picospritzer (General Valve) dispensed drug (dissolved in recording solution) onto the recorded neuron via a pressure ejection. Pipette location relative to the recorded cell, along with ejection pressure, were held constant throughout the recording. Ejection duration was varied to enable collection of quasi-concentration response curves.

### Nicotine pharmacokinetic modeling

Nicotine pharmacokinetic simulations in rats were conducted using MATLAB and SimBiology (MathWorks). A two-compartment model was built, simulating a central (blood, plasma, CSF, etc.) and peripheral (fat, muscle, and other poorly perfused tissues) compartment. Hereafter, “plasma” denotes the central compartment. Model parameters were selected based on previous studies (Kyerematen et al., 1988; Shivange et al., 2019). Key model parameters include: central compartment capacity (5 l/kg), elimination rate constant (0.8), forward rate constant (central->peripheral; 1.5), reverse rate constant (peripheral->central; 1.2), and clearance rate constant (1.4). Limitations include, but are not limited to, the following: (1) a standard 350-g rat was modeled, and (2) variability in nicotine metabolism across animals was not accounted for. Model parameters were first validated by comparing rat plasma nicotine kinetics following an arterial bolus dose (Kyerematen et al., 1988) to the predicted nicotine kinetics predicted by the model after simulating the same bolus dose into the central compartment. Moreover, the peak predicted plasma [nicotine] levels predicted by the model agree with other reports that measured rat nicotine levels in plasma after intravenous dosing (Craig et al., 2014). To model predicted plasma nicotine levels in our experimental rats, the model was enabled to accept infusion time vectors from SA sessions. The model returned a predicted [nicotine] versus time profile, which was used to calculate key metrics such as peak predicted plasma [nicotine] and predicted plasma area under the curve (AUC) for specified time periods after the start of the SA session. Predicted steady-state plasma [nicotine] levels (10–50 ng/ml) in our experimental rats during nicotine SA sessions were similar to plasma nicotine levels measured in human smokers during unrestricted cigarette smoking (Benowitz et al., 1983) and during regular, intermittent cigarette smoking (Isaac and Rand, 1972; Benowitz et al., 1982). In particular, we observed peaks and troughs in the predicted plasma nicotine versus time profile that were reminiscent of data from humans during intermittent cigarette smoking (Isaac and Rand, 1972).

### Experimental design and statistical analysis

**Figure 1.**
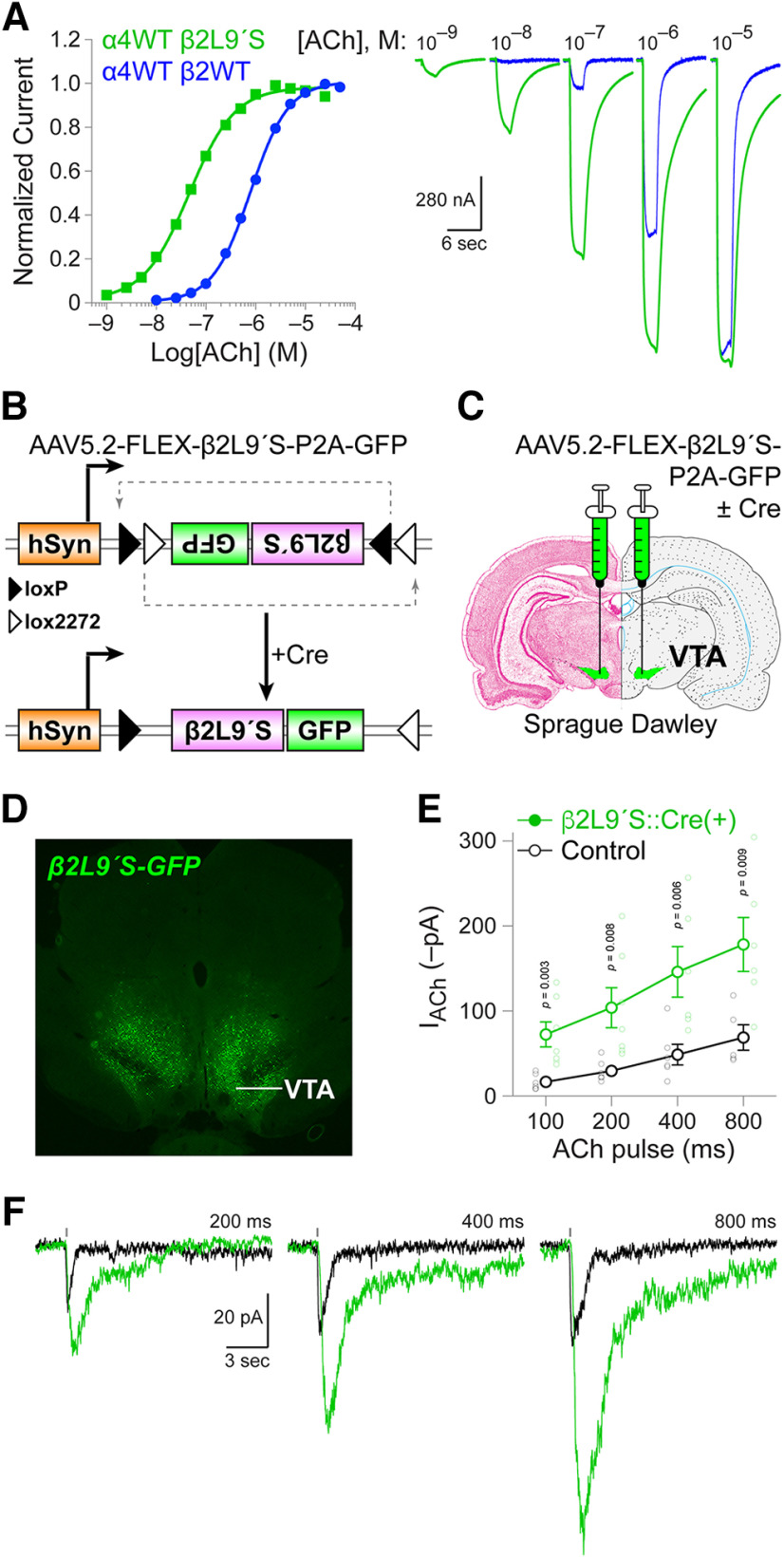
Creation and validation of β2 Leu9′Ser AAV. ***A***, β2 Leu9′Ser validation in *Xenopus laevis* oocytes. Oocytes were injected with rat nAChR subunit mRNAs: α4 (WT) and β2 (WT) or α4 (WT) and β2 (Leu9′Ser) in a α:β ratio of 1:2. ACh-evoked inward currents were recorded using two-electrode voltage clamp recordings, and the resulting concentration-response curve is shown for both groups. Representative traces for the indicated ACh concentrations are shown at right for oocytes injected with mRNAs for α4WT β2WT and α4WT β2Leu9′Ser. ***B***, Design of adeno-associated virus for Cre-dependent expression of (1) β2Leu9′Ser nAChR subunits and (2) GFP. ***C***, AAVs for expression of β2Leu9′Ser subunits (±Cre) were injected bilaterally into the VTA of SD rats. ***D***, Example β2Leu9′Ser GFP reporter expression. After injections described in ***C***, GFP expression was verified in VTA. ***E***, ***F***, Gain-of-function validation in β2Leu9′Ser rats. Brain slices containing the VTA were prepared from naive control rats or β2Leu9′Ser::Cre(+) rats, and patch clamp recordings were made from VTA neurons. ACh (300 μm) was applied to the recorded cell using a puffer pipette in voltage clamp mode. Mean (±SEM) inward current amplitude is shown (***E***), along with all responses from individual cells (small open circles). ***F***, Representative ACh (300 μm, pulse duration indicated) responses for control and β2Leu9′Ser::Cre VTA neurons.

**Figure 2.**
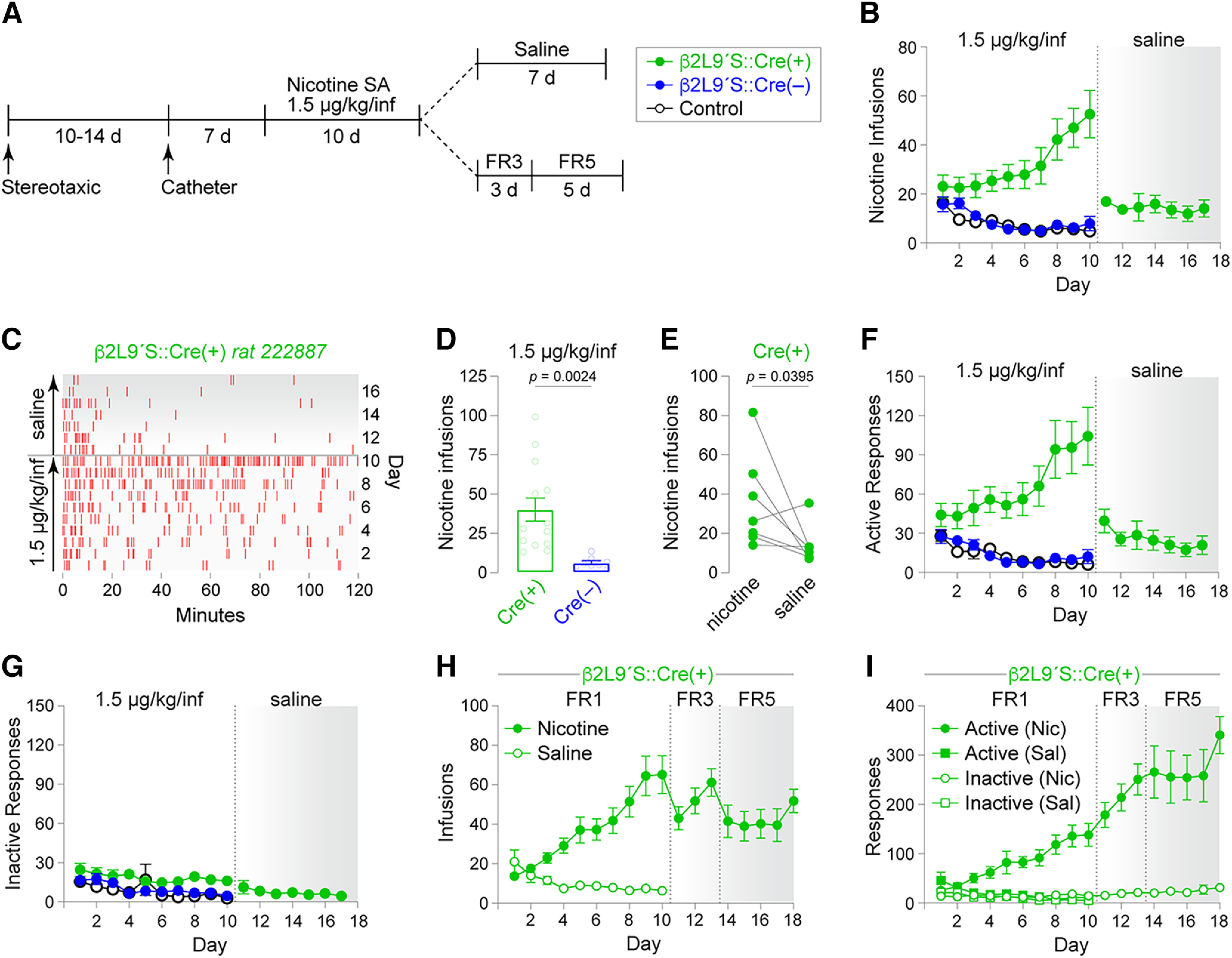
β2Leu9′Ser rats acquire nicotine SA at 1.5 μg/kg/infusion. ***A***, Experimental workflow. Rats were microinjected in VTA with β2Leu9′Ser AAVs, food trained, implanted with jugular catheters, and trained to self-administer nicotine (1.5 μg/kg/infusion; FR1) for 10 d. One subgroup of rats was switched to saline SA for an additional 7 d. A separate FR1 group was transitioned to nicotine SA with a response requirement of FR3 and then FR5. Naive SD rats served as an additional control. ***B***, Mean (±SEM) infusions for nicotine SA at 1.5 μg/kg/inf, followed by saline substitution (in *n* = 7 of 14 Cre(+) rats). ***C***, Representative 17 d infusion record for a β2 Leu9′Ser rat for 10-d nicotine (1.5 μg/kg/inf) SA and 7-d saline SA. ***D***, Nicotine infusions in β2Leu9′Ser::Cre(+) and Cre(−) rats for acquisition days 6–10. ***E***, Saline substitution in β2Leu9′Ser::Cre(+) rats. Average infusions per animal across days 6–10 before and after the switch to saline in β2Leu9′Ser::Cre(+) rats is shown. Active (***F***) and inactive (***G***) responses for infusion data shown in ***B***. ***H***, Mean (±SEM) infusions for nicotine SA at 1.5 μg/kg/inf at response requirements of FR1, FR3, and FR5 for the indicated days. Another group was trained to self-administer saline for 10 d. ***I***, Active and Inactive responses for infusion data shown in ***H***.

**Figure 3.**
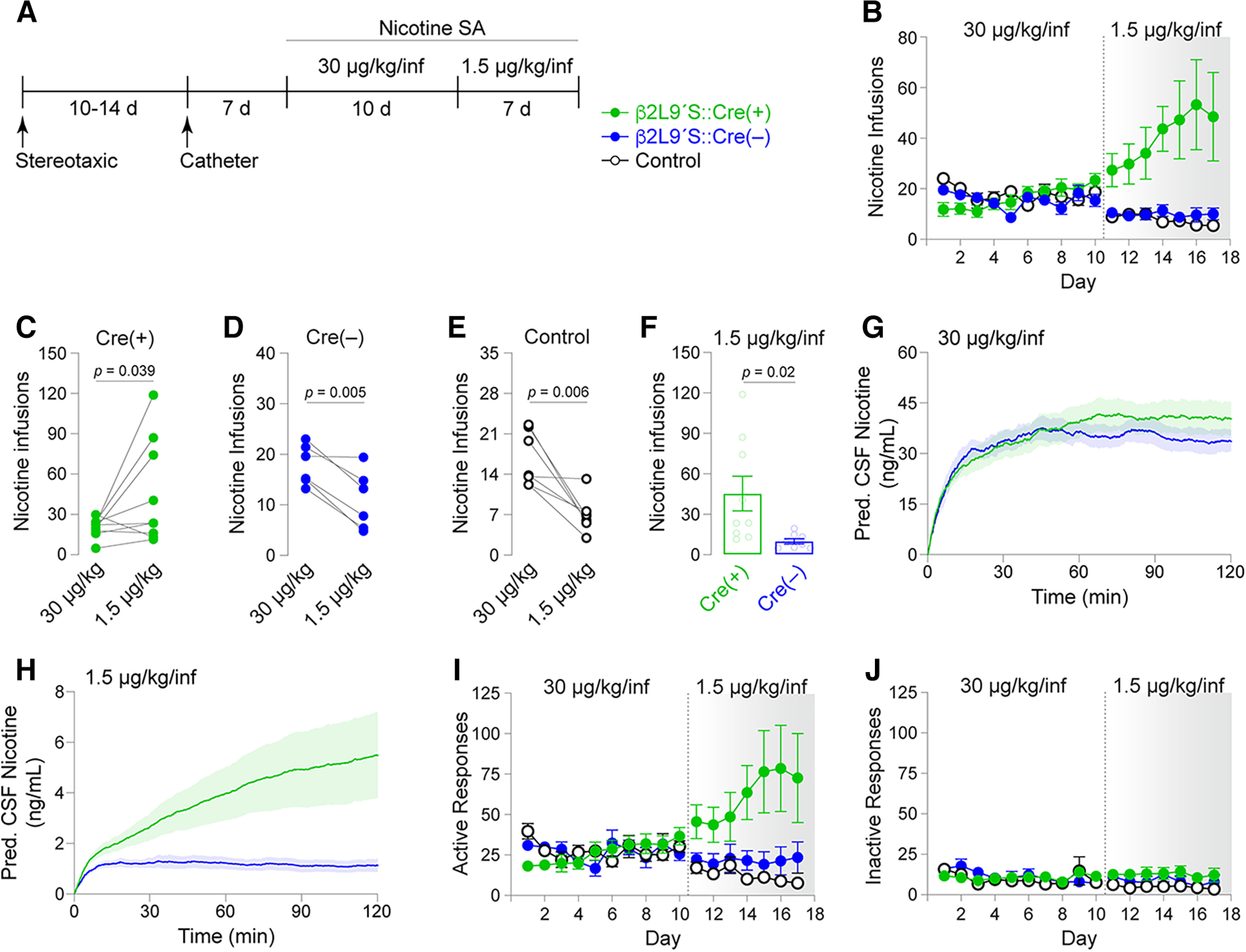
Subthreshold nicotine concentrations distinguish β2Leu9′Ser rats after standard nicotine SA. ***A***, Experimental workflow. Rats were microinjected in VTA with β2Leu9′Ser AAVs (±Cre), implanted with jugular catheters, and trained on nicotine SA (30 μg/kg/inf) for 10 d. Rats were then switched to 1.5 μg/kg/inf nicotine for 7 d. ***B***, Mean (±SEM) infusions nicotine SA at 30 μg/kg/inf, followed by 1.5 μg/kg/inf. ***C*–*E***, Reduced nicotine concentration enhances nicotine SA in β2Leu9′Ser::Cre(+) rats but reduces SA in controls. Average infusions per animal across days 6–10 before and after the switch to 1.5 μg/kg/inf (days 13–17) in (***C***) β2Leu9′Ser::Cre(+), (***D***) β2Leu9′Ser::Cre(−), and (***E***) SD control rats. ***F***, Nicotine infusions in β2Leu9′Ser::Cre(+) (*n* = 9) and Cre(−) (*n* = 6) rats during 1.5 μg/kg/inf after 10 d at 30 μg/kg/inf. ***G***, Predicted CSF nicotine during 30 μg/kg/inf nicotine SA (days 6–10) in β2Leu9′Ser::Cre(+) and Cre(−) rats. Nicotine infusion data from Figure 3*B* was used to estimate CSF nicotine levels (±SEM, shaded above/below) while rats self-administered 30 μg/kg/inf. ***H***, Predicted CSF nicotine in β2Leu9′Ser::Cre(+) and Cre(−) rats during SA of 1.5 μg/kg/inf. CSF nicotine was determined for days 13–17 as described above (***G***) for 30 μg/kg/inf. Active (***I***) and inactive (***J***) responses for infusion data shown in ***B***.

**Figure 4.**
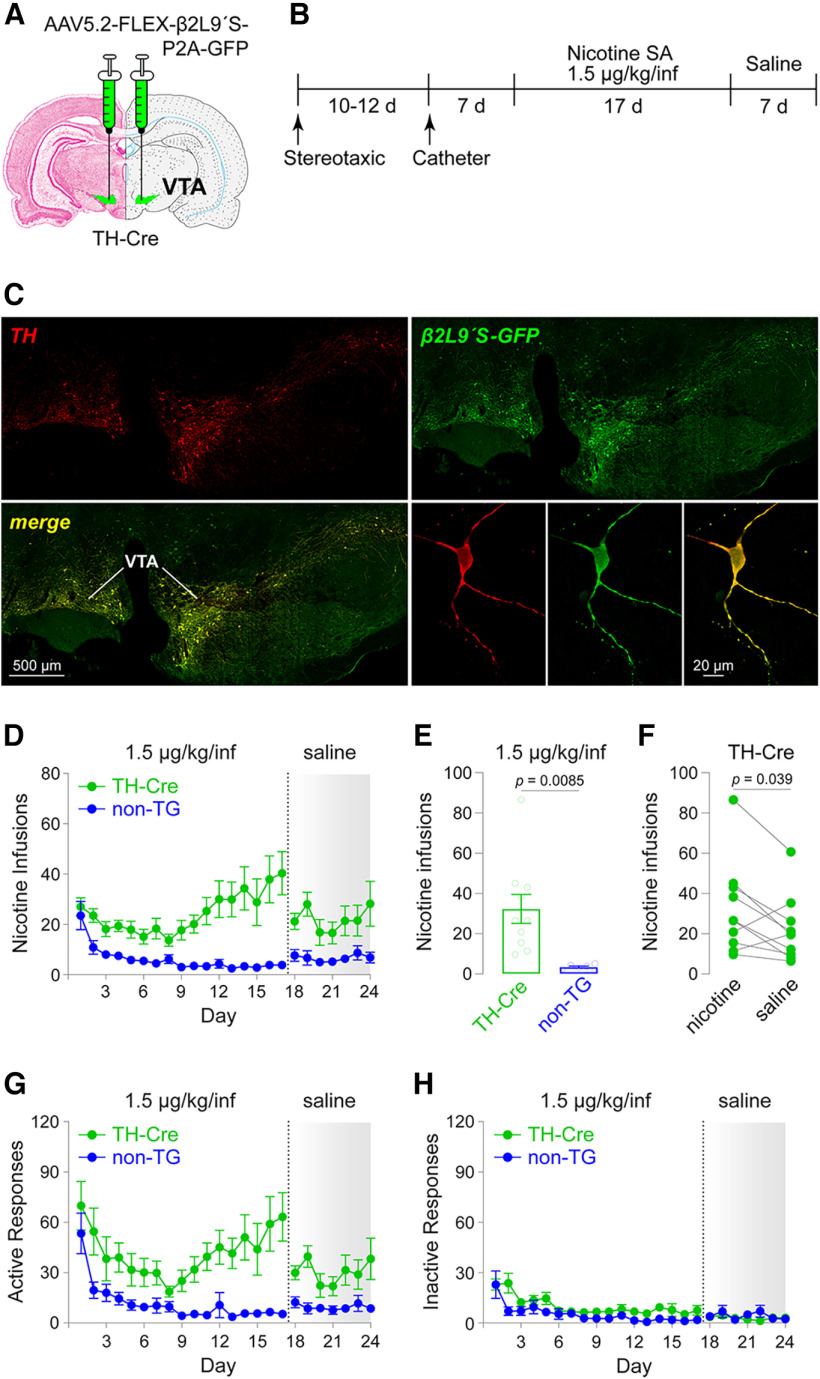
Hypersensitive β2* nAChRs in DA neurons: sensitized nicotine SA. ***A***, AAV5.2-FLEX-β2Leu9′Ser-P2A-GFP was bilaterally microinjected into the VTA of TH-Cre or littermate nontransgenic (non-Tg) rats. ***B***, Experimental workflow. Rats were microinjected in VTA (***A***), food trained, implanted with jugular catheters, and trained to self-administer nicotine (1.5 μg/kg/inf) for 17 d. Rats were then switched to saline SA for 7 d. ***C***, β2Leu9′Ser-P2A-GFP expression in TH(+) neurons. Four weeks after microinjection (***A***), VTA sections were co-stained for TH and GFP to confirm GFP expression in TH(+) neurons. Bottom right panels show a single TH neuron expressing β2Leu9′Ser-P2A-GFP. ***D***, Mean (±SEM) infusions nicotine SA (1.5 μg/kg/inf then saline). ***E***, Nicotine infusions (±SEM) in TH-Cre (*n* = 10) and non-Tg (*n* = 6) β2 Leu9′Ser rats during 1.5 μg/kg/inf. ***F***, Saline substitution reduces nicotine SA in TH-Cre β2 Leu9′Ser rats. Average infusions across days 11–17 before and after the switch to saline (days 18–24) are shown. Active (***G***) and inactive (***H***) responses for infusion data shown in ***D***.

**Figure 5.**
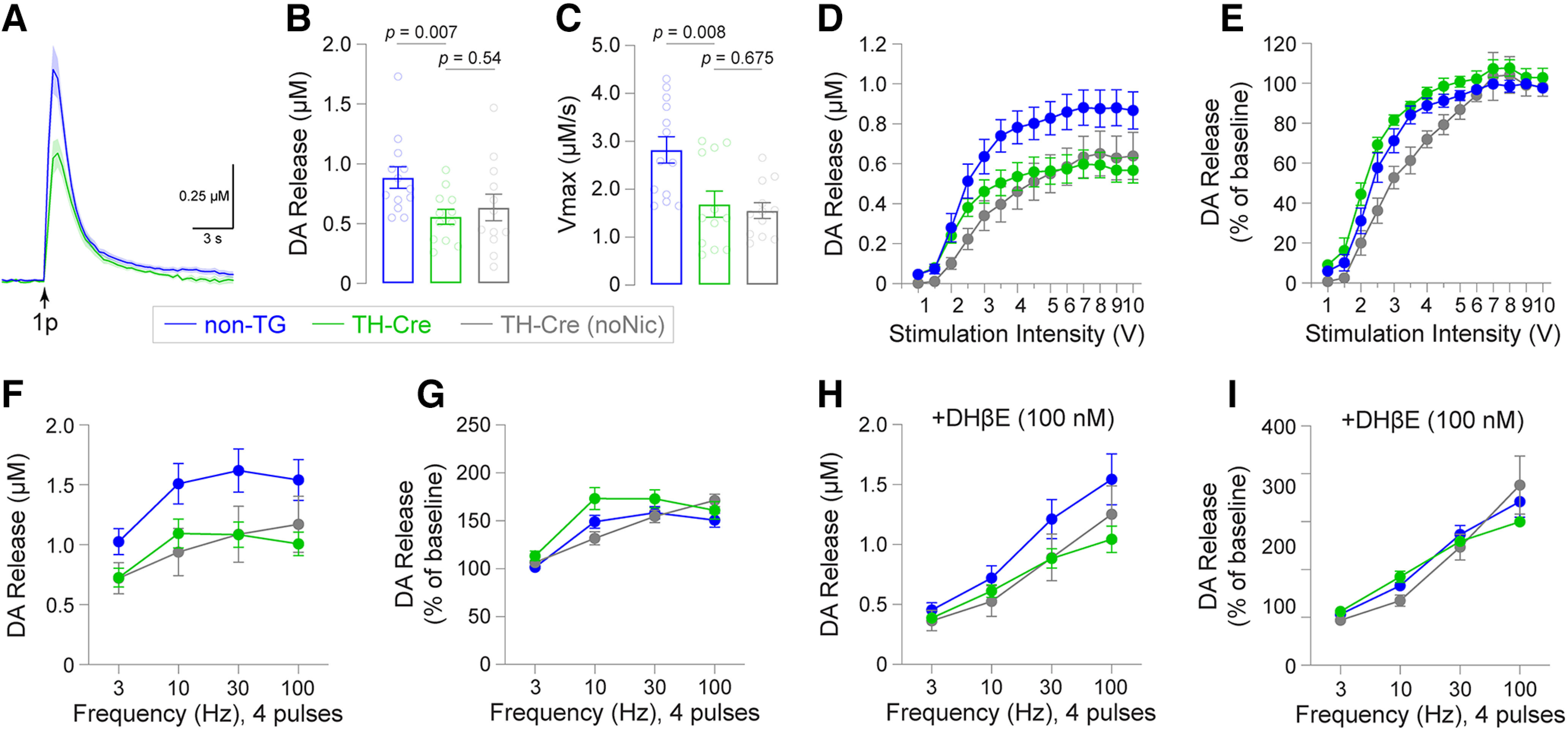
Altered DA release and uptake in TH-Cre β2Leu9′Ser rats with a history of nicotine SA. After nicotine SA (Fig. 4), NAc core slices were prepared from TH-Cre and non-Tg controls microinjected in VTA with AAV5.2-FLEX-β2Leu9′Ser-P2A-GFP. Another group of AAV5.2-FLEX-β2Leu9′Ser-P2A-GFP-injected TH-Cre animals, but which did not have exposure to nicotine, are included as an additional control. ***A***, Average (±SEM, shaded) DA release trace for the indicated groups. ***B***, ***C***, Electrically evoked (single pulse stimulation) DA release (***B***) and DA uptake (***C***). ***D***, ***E***, Input/output relationship for evoked DA release in TH-Cre and non-Tg rats. DA release from NAc core slices was measured at a range of stimulation intensities (0.5–10 V, single pulse), and is expressed as peak [DA] release (***D***) and normalized DA release I. ***F***, ***G***, DA release after four-pulse stimulation at 3, 10, 30, and 100 Hz is shown as peak [DA] (***F***) and expressed as a percentage of DA release evoked by single pulse stimulation (***G***). ***H***, ***I***, DhβE (100 nm) was bath-applied to the NAc core slices described above, and multipulse stimulation was conducted and reported as described for ***F***, ***G***.

**Figure 6.**
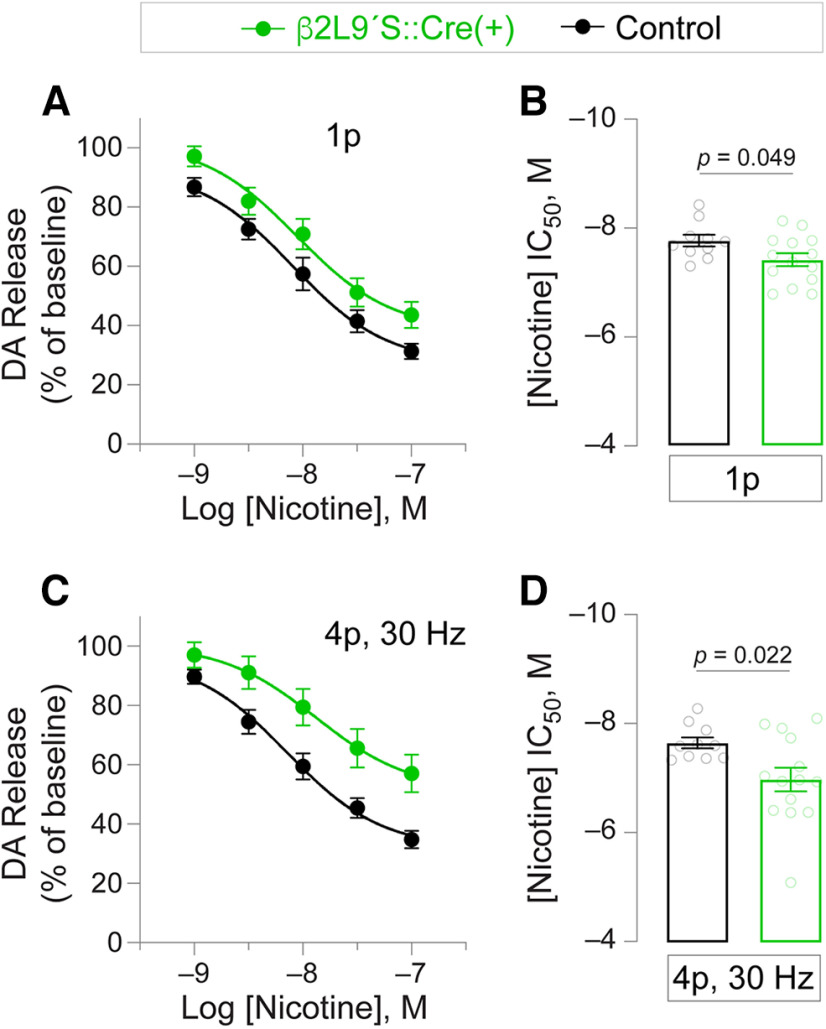
Modulation of DA release by nicotine in β2Leu9′Ser rats. ***A***, Mean DA release (±SEM) in response to single pulse stimulation is shown relative to baseline (no drug) release induced by single pulse stimulation. ***B***, Mean (±SEM) nicotine IC_50_ values for individual slices used to construct data in ***A***. ***C***, Mean DA release (±SEM) in response to multipulse (4p, 30 Hz) stimulation is shown relative to baseline (no drug) release induced by 4p, 30-Hz stimulation. ***D***, Mean (±SEM) nicotine IC_50_ values for individual slices used to construct data in ***C***.

SA data files, produced by Med Associates MedPC IV software, were analyzed with custom scripts written in MATLAB and/or GraphPad Prism 9. All sample sizes and results from statistical analyses for Figures 1-6 are presented in Table 1. Patch clamp and two-electrode voltage clamp data files were analyzed with custom MATLAB scripts. Scalable vector graphics were produced from MATLAB figures using functions written by Salva Ardid (https://github.com/kupiqu/fig2svg). Rat brain anatomy graphics were derived from “Brain Maps 4.0” (Larry Swanson; University of Southern California; Swanson, 2018).

**Table 1:** Statistical table

Figure	Parameter	Sample size	Statistical test	Significance level
1*E*	Current (−pA), 100-ms pulse	Control: 6 cells Cre(+): 7 cells	Unpaired *t* test (one-tailed)	*p *=* *0.003, *t *=* *3.386, df = 11
1*E*	Current (−pA), 200-ms pulse	Control: 6 cells Cre(+): 7 cells	Unpaired *t* test (one-tailed)	*p *=* *0.0079, *t *=* *2.848, df = 11
1*E*	Current (−pA), 400-ms pulse	Control: 6 cells Cre(+): 6 cells	Unpaired *t* test (one-tailed)	*p *=* *0.0064, *t *=* *3.026, df = 10
1*E*	Current (−pA), 800-ms pulse	Control: 5 cells Cre(+): 6 cells	Unpaired *t* test (one-tailed)	*p *=* *0.0088, *t *=* *2.901, df = 9
2*D*	Nicotine infusions across SA days 6–10	Cre(+): 14 rats Cre(−): 8 rats	Unpaired *t* test	*p *=* *0.0024, *t *=* *3.465, df = 20
2*E*	Nicotine infusions, SA days 6–10 vs days 13–17	*N* = 7 rats	Paired *t* test	*p *=* *0.0395, *t *=* *2.113, df = 6
3*C*	Nicotine infusions, SA days 6–10 vs days 13–17	Cre(+): 9 rats	Paired *t* test	*p *=* *0.0386, *t *=* *2.028, df = 8
3*D*	Nicotine infusions, SA days 6–10 vs day 13–17	Cre(−): 6 rats	Paired *t* test	*p *=* *0.0051, *t *=* *4.745, df = 5
3*E*	Nicotine infusions, SA days 6–10 vs days 13–17	Naive SD: 7 rats	Paired *t* test	*p *=* *0.0059, *t *=* *4.160, df = 6
3*F*	Nicotine infusions across SA days 13–17	Cre(+): 9 rats Cre(−): 8 rats	Unpaired *t* test	*p *=* *0.022, *t *=* *2.555, df = 15
4*E*	Nicotine infusions across SA days 11–17	TH-Cre: 10 rats Non-TG: 6 rats	Unpaired *t* test	*p *=* *0.0085, *t *=* *3.062, df = 14
4*F*	Nicotine infusions, SA days 11–17 vs day 18–24	TH-Cre: 10 rats	Paired *t* test	*p *=* *0.0391, *t *=* *2.412, df = 9
5*B*	Peak dopamine concentration (in μm)	TH-Cre: 12 slices from 3 rats Non-TG: 13 slices from 3 rats	Unpaired *t* test	*p *=* *0.007, *t *=* *2.956, df = 23
5*B*	Peak dopamine concentration (in μm)	TH-Cre (noNic): 12 slices from 3 rats Non-TG: 13 slices from 3 rats	Unpaired *t* test	*p *=* *0.091, *t *=* *1.763, df = 23
5*C*	V_max_ (μm/s)	TH-Cre: 12 slices from 3 rats Non-TG: 13 slices from 3 rats	Unpaired *t* test	*p *=* *0.0079, *t *=* *2.91, df = 23
5*C*	V_max_ (μm/s)	TH-Cre (noNic): 12 slices from 3 rats Non-TG: 13 slices from 3 rats	Unpaired *t* test	*p *=* *0.0008, *t *=* *3.865, df = 23
5*F*	DA release (μm), 3-Hz multipulse	TH-Cre: 12 slices from 3 rats Non-TG: 13 slices from 3 rats	Unpaired *t* test	*p *=* *0.038, *t *=* *2.202, df = 23
5*F*	DA release (μm), 30-Hz multipulse	TH-Cre: 12 slices from 3 rats Non-TG: 13 slices from 3 rats	Unpaired *t* test	*p *=* *0.0197, *t *=* *2.507, df = 23
5*F*	DA release (μm), 100-Hz multipulse	TH-Cre: 12 slices from 3 rats Non-TG: 13 slices from 3 rats	Unpaired *t* test	*p *=* *0.0148, *t *=* *2.634, df = 23
5*G*	Normalized DA release, 10 Hz	TH-Cre: 12 slices from 3 rats TH-Cre (noNic): 12 slices from 3 rats	Unpaired *t* test	*p *=* *0.0053, *t *=* *3.093, df = 22
6*B*	1p stimulation, DA release nicotine IC_50_	Cre(+): 14 slices from 4 rats Naive SD: 10 slices from 4 rats	Unpaired *t* test	*p *=* *0.0488, *t *=* *2.086, df = 22
6*D*	4p (30 Hz) stimulation, DA release nicotine IC_50_	Cre(+): 14 slices from 4 rats Naive SD: 10 slices from 4 rats	Unpaired *t* test	*p *=* *0.0223, *t *=* *2.459, df = 22

## Results

### Hypersensitive β2 nAChR expression

Because most neuronal nAChRs have similar agonist sensitivities, no agonists have been reported that can be delivered to a behaving animal to induce strong (i.e., full agonism) and specific (i.e., no other subtypes affected) activation of β2 nAChRs. Based on prior work, substitution of a serine at the 9′ leucine residue in the second transmembrane α-helix is expected to increase receptor sensitivity sufficiently to allow low concentrations of agonists to be used for selective activation of only those receptors harboring the Leu9′ mutation (Labarca et al., 1995, 2001; Fonck et al., 2003, 2009; Lester et al., 2003; Orb et al., 2004; Tapper et al., 2004; Drenan et al., 2008, 2010; Cohen et al., 2012; Engle et al., 2012, 2013, 2015; Wang et al., 2014). To validate the Leu9′ mutation in the β2 subunit, we injected *Xenopus* oocytes with WT α4 and Leu9′Ser β2 mRNAs and recorded ACh-evoked currents using two-electrode voltage clamp. As expected, oocytes injected with α4β2Leu9′Ser nAChRs (EC_50_: 46 nm; 95% CI: 44–47 nm) were more sensitive to ACh than control α4β2 nAChRs (EC_50_: 788 nm; 95% CI: 774–802 nm; Fig. 1*A*). We produced an AAV for expression of β2Leu9′Ser nAChR subunits and a soluble GFP marker. Cre recombinase co-expression is co-injected to flip the β2-P2A-GFP cassette into the correct orientation relative to the human synapsin promoter (Fig. 1*B*). We tested this vector by infusing it, together with an AAV driving Cre expression, into the ventral midbrain of adult SD rats (Fig. 1*C*). This resulted in robust GFP expression in the VTA (Fig. 1*D*). Although this approach does not use cell-specific promoters to restrict transgene expression, functional expression of heteromeric nAChRs harboring the β2Leu9′Ser subunit is nonetheless restricted to neurons that also express α4 and/or α6 subunits. These subunits are highly enriched in VTA DA, GABA, and glutamate neurons relative to adjacent brain areas (Azam et al., 2002; Yan et al., 2018). To confirm that this AAV induced a β2 nAChR gain-of-function, we recorded ACh-evoked inward currents in VTA neurons in brain slices from control and β2Leu9′Ser::Cre(+) rats. At all ACh pulse durations, inward currents in VTA neurons from β2Leu9′Ser::Cre(+) rats were significantly larger than in control rats (100 ms, *p *=* *0.003, one-tailed *t* test, *t *=* *3.386, df = 11; 200 ms, *p *=* *0.008, one-tailed *t* test, *t *=* *2.848, df = 11; 400 ms, *p *=* *0.006, one-tailed *t* test, *t *=* *3.026, df = 10; 800 ms, *p *=* *0.009, one-tailed *t* test, *t *=* *2.901, df = 9; Fig. 1*E*,*F*).

### β2Leu9′Ser rats acquire nicotine SA

To determine whether β2 nAChR activation in the VTA is sufficient for acquisition of nicotine SA, SD rats were infused with β2Leu9′Ser and Cre vectors in the VTA via stereotaxic surgery. Indwelling jugular catheters were placed in a subsequent surgery (Fig. 2*A*). Rats were tested for acquisition of nicotine SA using a dose of 1.5 μg/kg/infusion, which is 20-fold lower than the standard training dose (30 μg/kg/inf). After 10 nicotine SA sessions, vehicle (saline) was substituted for nicotine in (*n* = 7 of 14) of Cre(+) rats to determine whether nicotine (at 1.5 μg/kg/inf) was reinforcing (Fig. 2*B*). Control groups included (1) rats infused with β2Leu9′Ser vectors but without Cre vectors (*n* = 8), and (2) naive SD male rats (*n* = 9). Whereas these two control groups did not acquire nicotine SA at 1.5 μg/kg/inf, 14 of 22 β2Leu9′Ser rats with Cre co-expression exhibited acquisition of nicotine SA (Fig. 2*B*). Eight of 22 β2Leu9′Ser::Cre(+) rats failed to maintain a minimum number of infusions and were considered to have failed to acquire SA. Saline substitution after 10 nicotine SA sessions reduced nicotine self-infusions (Fig. 2*B*), suggesting that β2Leu9′Ser::Cre(+) rats were responding to 1.5 μg/kg nicotine because it was reinforcing. A complete infusion record is shown for nicotine SA and saline substitution for a representative β2Leu9′Ser::Cre(+) rat (Fig. 2*C*). The mean number of nicotine infusions across sessions 6–10 was significantly greater for β2Leu9′Ser rats that received Cre recombinase compared with the Cre(−) group (*p *=* *0.0024, unpaired *t* test, *t *=* *3.465, df = 20; Fig. 2*D*). Saline substitution reduced nicotine self-infusions in β2Leu9′Ser::Cre(+) rats (*p *=* *0.0395, paired *t* test, *t *=* *2.113, df = 6; Fig. 2*E*). Active responses largely mimicked the infusion data (Fig. 2*F*), and none of the three groups had notable responding on the inactive nose poke (Fig. 2*G*).

To provide additional evidence that β2Leu9′Ser::Cre(+) rats were responding for 1.5 μg/kg nicotine, two additional control experiments were performed. First, nicotine-naive β2Leu9′Ser::Cre(+) rats were trained to self-administer saline for 10 sessions. Responding for saline declined (Fig. 2*H*,*I*), similar to β2Leu9′Ser::Cre(−) and SD control rats self-administering 1.5 μg/kg/inf nicotine, confirming that β2Leu9′Ser::Cre(+) rats were responding for nicotine at 1.5 μg/kg/inf. Second, after 10 SA sessions at FR1, β2Leu9′Ser::Cre(+) rats self-administering 1.5 μg/kg/inf nicotine were transitioned to a response requirement of FR3 and then FR5. Infusions were maintained after switching rats to FR3 and appeared to decline only slightly, then stabilize, at FR5 (Fig. 2*H*). Active responses increased during FR3 and FR5 compared with FR1, with no apparent increase in responses on the inactive nose poke (Fig. 2*I*). These additional controls confirm that β2Leu9′Ser::Cre(+) rats responded specifically for 1.5 μg/kg/inf nicotine.

Next, we sought additional evidence that nicotine SA at a dose of 1.5 μg/kg/inf is reinforcing for rats expressing VTA β2Leu9′Ser nAChRs. We tested the hypothesis that, after acquiring nicotine SA at the standard training dose (30 μg/kg/inf), expression of β2Leu9′Ser nAChRs in VTA would enable nicotine SA to be maintained following a switch to 1.5 μg/kg/inf. As above, three groups of SD rats were tested: (1) β2Leu9′Ser::Cre(+) (*n* = 10), (2) β2Leu9′Ser::Cre(−) (*n* = 6), and (3) naive SD rats (*n* = 7). After stereotaxic and jugular catheter surgery, rats were allowed to self-administer 30 μg/kg/inf nicotine on an FR1 schedule of reinforcement for 10 d (Fig. 3*A*). The number of infusions and active/inactive responses for the naive SD rats and β2Leu9′Ser::Cre(−) rats was as expected and consistent with recent nicotine SA work from our lab that employed identical acquisition conditions (Fig. 3*B*). β2Leu9′Ser::Cre(+) rats (*n* = 8 of 10) acquired nicotine IVSA at 30 μg/kg/inf very similarly to both control groups (Fig. 3*B*). *N* = 2 (of 10) β2Leu9′Ser::Cre(+) rats, *n* = 2 (of 8) β2Leu9′Ser::Cre(−) rats, and *n* = 0 (of 7) naive SD control rats failed to acquire nicotine SA. When we substituted 1.5 μg/kg/inf (the lower nicotine concentration used in Fig. 2) for 30 μg/kg/inf (Fig. 3*A*), β2Leu9′Ser::Cre(+) rats showed a trend toward increased infusions and active responses (Fig. 3*B*). Notably, the two control groups reduced their infusions when the dose was reduced to 1.5 μg/kg/inf. Within-subject comparisons confirmed that β2Leu9′Ser::Cre(+) rats significantly increased their number of infusions following reduction of the nicotine dose to 1.5 μg/kg/inf (*p *=* *0.0386, paired *t* test, *t *=* *2.028, df = 8; Fig. 3*C*). By contrast, β2Leu9′Ser::Cre(−) (*p *=* *0.0051, paired *t* test, *t *=* *4.745, df = 5; Fig. 3*D*) and naive SD control (*p *=* *0.0059, paired *t* test, *t *=* *4.160, df = 6; Fig. 3*E*) rats significantly reduced their nicotine infusions following the switch from 30 μg/kg/inf to 1.5 μg/kg/inf. Mean nicotine (1.5 μg/kg/inf, days 13–17) infusions were significantly greater for β2Leu9′Ser::Cre(+) rats compared with β2Leu9′Ser::Cre(−) controls (*p *=* *0.022, unpaired *t* test, *t *=* *2.555, df = 15; Fig. 3*F*). Modeled/predicted CSF nicotine concentrations were similar between β2Leu9′Ser::Cre(+) rats compared with β2Leu9′Ser::Cre(−) controls self-administering 30 μg/kg/inf (Fig. 3*G*), but β2Leu9′Ser::Cre(+) rats self-administering 1.5 μg/kg/inf exhibited greater predicted nicotine levels compared with controls (Fig. 3*H*). Active responses largely reflected the infusion data (Fig. 3*I*), and inactive responses were low and unremarkable (Fig. 3*J*).

### DA neuron nAChR activation and nicotine SA

The results from SD rats involve virus injections into VTA, but they do not select for a specific VTA cell type (DA, GABA, glutamate, etc.). Although a majority of VTA neurons are dopaminergic, our results do not rule out the possibility that VTA GABA or other cell types mediate the behavioral response we reported. Next, we tested the hypothesis that activation of β2* nAChRs on VTA DA neurons is sufficient for nicotine reinforcement. Transgenic Long–Evans rats that express Cre recombinase in tyrosine hydroxylase (TH) neurons (*n* = 11; Witten et al., 2011) were infused with our Cre-dependent β2Leu9′Ser vector in the VTA via stereotaxic surgery (Fig. 4*A*), catheterized, and allowed to acquire nicotine SA at a nicotine dose of 1.5 μg/kg/inf (Fig. 4*B*). Expression in TH-Cre rats resulted in strong GFP reporter expression in neurons that could be co-labeled with anti-TH antibody staining (Fig. 4*C*). Nontransgenic (non-Tg) littermates of TH-Cre rats (*n* = 6), infused in the VTA with the same Cre-dependent β2Leu9′Ser vector, served as controls. *N* = 10 of 11 injected TH-Cre β2Leu9′Ser rats acquired SA (*n* = 1 of 11 failed acquisition), maintained 15–20 infusions per day through the first 10 d, then increased their mean self-infusions to ∼40 infusions by the 17th day (Fig. 4*D*). By contrast, non-Tg rats microinjected in the VTA with the β2Leu9′Ser vector had very few infusions (<5/d) through day #17 (Fig. 4*D*). Indeed, TH-Cre rats earned significantly more infusions than non-Tg littermates during days 11–17 (*p *=* *0.0085, unpaired *t* test, *t *=* *3.062, df = 14; Fig. 4*E*). To confirm that 1.5 μg/kg/inf nicotine is reinforcing, saline was substituted for nicotine for days 18–24 (Fig. 4*D*). Compared with their presaline level of self-infusions, TH-Cre β2Leu9′Ser rats significantly reduced their infusions when saline was substituted for nicotine (*p *=* *0.0391, paired *t* test, *t *=* *2.412, df = 9; Fig. 4*F*). Active responses largely reflected the infusion data (Fig. 4*G*), and inactive responses were very low (Fig. 4*H*).

### DA release in β2Leu9′Ser rats

Because SA of other psychostimulants (i.e., cocaine) induces changes to dopamine transporter function and evoked dopamine release (Calipari et al., 2013), we asked whether TH-Cre β2Leu9′Ser rats (*n* = 3) with and without a history of nicotine SA exhibited differential DA dynamics compared with non-Tg rats (*n* = 3). Following SA, brain slices were prepared containing NAc core (NAcc) and fast scan cyclic voltammetry was used to record electrically-evoked DA release. Peak [DA] achieved by single pulse stimulation (representative traces; Fig. 5*A*) was significantly reduced in TH-Cre β2Leu9′Ser NAcc slices compared with non-Tg controls (*p *=* *0.007, unpaired *t* test, *t *=* *2.956, df = 23; Fig. 5*B*). Nicotine history did not appear to account for this effect in the TH-Cre group, as there was no significant difference in DA release between nicotine-experienced TH-Cre and nicotine-naive TH-Cre rats (Fig. 5*B*). DA uptake (V_max_) was slower in TH-Cre (*p *= 0.0079, unpaired *t* test, *t *=* *2.91, df = 23) rats compared with non-Tg controls, which was also not dependent on nicotine history (Fig. 5*C*). Results in Figure 5*A–C* were collected with our standard stimulation intensity of 7.5 V input (350-μA output to the slice). To determine whether reduced DA release in TH-Cre β2Leu9′Ser NAcc was dependent on stimulation intensity, we measured single-pulse evoked DA release at a range of stimulation intensities. Peak DA release in TH-Cre β2Leu9′Ser NAcc was systematically lower for stimulation input intensities from 3–10 V (Fig. 5*D*), which was not accounted for by nicotine history. Normalized release was similar to controls (Fig. 5*E*). Next, we determined whether DA release elicited under “phasic-like” stimulation differed in TH-Cre β2Leu9′Ser slices by using a four-pulse stimulation train across varying frequencies. Peak DA release evoked by a four-pulse stimulation train was reduced in TH-Cre β2Leu9′Ser NAcc at 3 Hz (*p *=* *0.038, *t *=* *2.202, df = 23), 30 Hz (*p *=* *0.0197, *t *=* *2.507, df = 23), and 100 Hz (*p *=* *0.0148, *t *=* *2.634, df = 23; Fig. 5*F*), although normalized DA release in TH-Cre β2Leu9′Ser NAcc was similar to non-Tg controls (Fig. 5*G*). Phasic-like DA release from TH-Cre β2Leu9′Ser slices was mostly unaffected by nicotine history, though there was a significant difference in normalized release at 10-Hz stimulation between TH-Cre and TH-Cre (nicotine naive; *p *=* *0.0053, unpaired *t* test, *t *=* *3.093, df = 22; Fig. 5*G*). Finally, we examined DA release in TH-Cre β2Leu9′Ser NAcc and non-Tg controls following blockade of β2* nAChRs with dihydro-β-erythroidine (DHβE, 100 nm). DA release evoked by a single stimulation was 51.8 ± 2.6% of predrug levels in TH-Cre β2Leu9′Ser NAcc and 45.0 ± 3.0% of predrug levels in non-Tg controls (*p *=* *0.1032, *t *=* *1.697, df = 23). DHβE was more effective at reducing DA release following low-frequency (3 and 10 Hz) stimulation trains compared with higher frequency trains (Fig. 5*H*). Normalized release was very similar for TH-Cre β2Leu9′Ser rats versus non-Tg control rats (Fig. 5*I*).

Finally, we asked whether DA release was differentially modifiable by nicotine in NAcc slices from β2Leu9′Ser::Cre(+) SD rats versus naive SD control rats. In control slices, nicotine reduced DA release evoked by single-pulse stimulation and a four-pulse train in a concentration-dependent manner (Fig. 6*A*,*C*). DA release from β2Leu9′Ser::Cre(+) NAcc was more resistant across these nicotine concentrations, resulting in an increased IC_50_ for single pulse stimulation and four-pulse train stimulation (Fig. 6*B*,*D*).

## Discussion

In this study, we demonstrate that expression of a gain-of-function β2 nAChR subunit in the ventral midbrain (VTA) of male rats is sufficient to significantly alter intravenous nicotine SA. Specifically, β2Leu9′Ser VTA expression enables rats to acquire operant responding for nicotine infusions at a dose that is 20-fold lower than the typical training dose. Additional experiments suggest that β2Leu9′Ser VTA expression may shift the dose response curve for nicotine reinforcement to the left. SA experiments in TH-Cre indicate that nAChR activation on VTA DA neurons is sufficient for nicotine reinforcement. β2Leu9′Ser behavioral alterations were accompanied by modifications to electrically evoked DA release. Together, these results contribute novel mechanistic insights into nicotine reinforcement mechanisms and cholinergic modulation of DA transmission.

### Nicotine reinforcement: VTA β2 nAChRs are sufficient

Loss-of-function studies using β2 nAChR antagonists (Corrigall et al., 1994) or β2 knock-out mice (Maskos et al., 2005; Pons et al., 2008; Tolu et al., 2013) have linked β2-containing nAChRs with nicotine reinforcement, while others (le Novère et al., 1999; King et al., 2004; Walters et al., 2006; Drenan et al., 2008, 2010; Naudé et al., 2016) have linked nicotine-related phenotypes (place preference, locomotor activation, motivation, etc.) with VTA β2-containing nAChRs. Notably, two prior studies served as a key starting point on which our study builds. Changeux and colleagues reported that re-expression of β2 subunits in the VTA of β2 KO mice was sufficient to restore intracranial (intra-VTA) self-administration of nicotine (Maskos et al., 2005). Tolu and colleagues later reported that this restoration of intracranial nicotine self-injection required re-expression of β2 in both DA and GABA neurons in VTA (Tolu et al., 2013). Our viral-mediated approach in adult rats overcomes several limitations of these important papers, including (1) examination of intravenous nicotine self-administration with saline substitution control experiments capable of detecting nicotine reinforcement, (2) examination of a species (rats) with nicotine metabolism that more closely approaches human nicotine metabolism (Matta et al., 2007), and (3) examination of experimental animals without preexisting global β2 KO that may cause developmental alterations to reward and/or learning-related circuits. For example, and related to the latter point, global β2 KO mice have altered passive avoidance learning (Picciotto et al., 1995; King et al., 2003). This study, by examining the effect of selective VTA β2 nAChR activation, provides a complementary approach to loss-of-function experiments.

Early work in rats established 30 μg/kg/inf as an optimal training dose for acquisition of nicotine SA (Corrigall and Coen, 1989; Donny et al., 1995). The dose of nicotine (1.5 μg/kg/inf) that supported acquisition of SA in rats expressing β2Leu9′Ser nAChRs is markedly lower than doses that normally support SA. This sensitization to low nicotine concentrations is further apparent when considering that CSF nicotine levels in rats self-administering 1.5 μg/kg/inf are predicted to be only ∼5 ng/ml after a 2-h SA session. CSF nicotine in untargeted control rats or β2Leu9′Ser-expressing rats is predicted to stabilize at 30–50 ng/ml during a 30 μg/kg/inf SA session, which is very similar to levels achieved in humans after cigarette smoking (Isaac and Rand, 1972). In 30 μg/kg/inf SA sessions, the predicted CSF versus time profile clearly suggests loading and maintenance behavior (Allain et al., 2015); rats robustly self-administer nicotine within the first 10–20 min, then slow their rate of nicotine self-injections to maintain predicted CSF levels between 30 and 50 ng/ml. β2Leu9′Ser-expressing rats self-administering 1.5 μg/kg/inf show a somewhat different profile, with a less-pronounced loading phase and stronger boosting of predicted CSF nicotine levels later in the 2-h SA session.

VTA neurons send projections to a variety of brain areas, including NAc, amygdala, prefrontal cortex, hippocampus, and lateral habenula (Lammel et al., 2008; Stamatakis et al., 2013). These neurons include DAergic, GABAergic, glutamatergic, and dual-transmitter cells (Yamaguchi et al., 2007, 2015; Lammel et al., 2014). β2-containing nAChRs are expressed both in somatodendritic and presynaptic compartments (Drenan et al., 2008, 2010), and systemic nicotine from IVSA likely acts on these presynaptic and/or postsynaptic β2Leu9′Ser nAChR populations to promote reinforcement. Chief among these, based on our results in TH-Cre rats, is the mesolimbic DA pathway that includes, though may not be limited to, VTA to NAc circuits. We noted that, on average, TH-Cre rats expressing β2Leu9′Ser in DA neurons show steady nicotine SA until day ∼10 when they escalate their intake. SD rats expressing β2Leu9′Ser nAChRs in all VTA neuron types appear to show a briefer latency to this escalatory behavior. There could be several explanations for this, such as: (1) expression in SD rats may have allowed a greater fraction of VTA neurons to be infected, resulting in more rapid acquisition of nicotine SA; (2) β2Leu9′Ser expression in DA and non-DA (GABA, glutamate, etc.) neurons in SD rats may have invoked additional circuitry that promotes acquisition of nicotine SA. Consistent with the latter, Maskos and colleagues reported that re-expression of β2 subunits only in VTA DA or GABA neurons in the VTA of β2 nAChR KO mice is insufficient to restore intra-VTA nicotine self-infusion behavior (Tolu et al., 2013). This suggested that the concerted action of β2 nAChRs on DA and GABA neurons are necessary for nicotine reinforcement.

### nAChR pore mutations and desensitization

Before being expressed *in vivo*, nAChR subunits with mutations in pore-lining residues (especially the 9′ and 13′ positions) were thoroughly studied *in vitro* with a variety of ectopic expression techniques. Cys-loop receptor subunits with 9′ and 13′ mutations exhibited increases in receptor sensitivity, elimination of inward rectification, and/or changes to single channel properties. These include increased probability of opening and stabilization of the open state (Revah et al., 1991; Bertrand et al., 1992; Yakel et al., 1993; Filatov and White, 1995; Labarca et al., 1995). These and other studies highlighted a key change in nAChR biophysics when the Leu9′ residue is modified: slowing or elimination of desensitization. Accordingly, nAChRs incorporating β2Leu9′Ser subunits likely have reduced desensitization when nicotine or ACh is bound. Instead of the normal shutoff of inward current flow in response to desensitization, a large fraction of the β2Leu9′Ser nAChR receptor pool likely retains activity while nicotine is available. Two results in this report, which should be further studied, are consistent with this: (1) inward current responses in VTA neurons from rats expressing β2Leu9′Ser subunits appeared to take longer to return to baseline after a pulse of ACh is delivered (Fig. 1*F*), and (2) greater concentrations of nicotine are required to attenuate (presumably via receptor desensitization) electrically-evoked DA release in NAcc slices from β2Leu9′Ser rats (Fig. 6). Whereas nicotine concentrations that are normally reinforcing have a rewarding and aversive component because of the balance of activation/desensitization, nicotine’s aversive property may be substantially reduced in rats expressing β2Leu9′Ser nAChR subunits in the VTA. Our approach, which can be conceptualized as a knock-down of VTA β2* nAChR desensitization, suggest that nicotine-mediated desensitization of β2-containing receptors plays an important role in the behavioral pharmacology of nicotine reward and reinforcement. By attenuating desensitization of mesolimbic β2* nAChRs, nicotine is transformed into a much more potent psychostimulant.

### DA release and β2Leu9′Ser nAChRs

In TH-Cre rats expressing β2Leu9′Ser nAChRs in VTA, the decrease in evoked DA release amplitude and the reduced DA uptake speed resembles previous results that examined DA release after cocaine SA. Calipari and colleagues reported that five cocaine SA sessions (6-h session, FR1, 1.5 mg/kg/inf, 40 infusion max) were sufficient to significantly reduce single pulse evoked DA release amplitude and velocity of uptake (Calipari et al., 2014). Phillips and colleagues reported that diminished DA signaling is correlated with escalation of cocaine SA, and that boosting DA via L-DOPA reverses escalation (Willuhn et al., 2014). These data suggest that β2Leu9′Ser nAChR subunit expression in VTA neuron somata and/or NAc presynaptic terminals induces neuroadaptations that blunt DA signaling while enhancing nicotine intake behavior. These putative adaptations are at least partially independent of nicotine exposure, as DA release in NAcc of nicotine naive rats expressing β2Leu9′Ser nAChR subunits is similar to release from β2Leu9′Ser rats with a history of nicotine exposure (Fig. 5). Normalized DA release, only at a 10-Hz stimulation frequency, was elevated in nicotine-experienced β2Leu9′Ser rats compared with nicotine-naive rats (Fig. 5*G*), but this may be because of sampling variation. Many adaptations can explain the blunted DA release phenomenon in β2Leu9′Ser NAcc, such as changes to DA transporter levels/function, changes in presynaptic Ca^2+^ handling, modulation of vesicular monoamine transporter, modified presynaptic release machinery, altered ACh release from cholinergic interneurons, etc. Future experiments will be required to understand how these adaptations enable rats to acquire and maintain nicotine SA at very low doses of nicotine. Interestingly, relative frequency-dependent increases in DA release are preserved in TH-Cre rats expressing β2Leu9′Ser nAChRs, although absolute DA release is reduced.

### Limitations and future studies

Our approach to targeting the mesolimbic pathway in SD rats includes viral transduction of VTA neurons, but we cannot exclude the possibility that nearby circuits also contribute. DA and/or GABA neurons in the adjacent substantia nigra (pars compacta and pars reticulata) may have been infected, potentially altering nicotine-related behaviors. However, this risk is mitigated by our data from TH-Cre rats; we would not have expected the SA results from TH-Cre rats to corroborate the results from SD rats if β2Leu9′Ser nAChR activation in non-DA neurons plays a role.

Expression of β2Leu9′Ser nAChR subunits in mesolimbic circuitry will provide several opportunities for future mechanistic research. For example, it will enable us to determine whether reinstatement of nicotine seeking occurs after acquisition of nicotine SA purely through the mesolimbic pathway. Moreover, our approach can also be used to determine whether activation of β2* nAChRs on other circuits is sufficient to support nicotine SA. Rats expressing β2Leu9′Ser nAChR subunits in the VTA could also be used in drug discovery efforts to determine whether lead compounds have abuse liability via activation of β2* nAChRs.

## Synthesis

Reviewing Editor: Christie Fowler, University of California, Irvine Decisions are customarily a result of the Reviewing Editor and the peer reviewers coming together and discussing their recommendations until a consensus is reached. When revisions are invited, a fact-based synthesis statement explaining their decision and outlining what is needed to prepare a revision will be listed below. The following reviewer(s) agreed to reveal their identity: Marina Picciotto.

Two experts in the field reviewed this manuscript, and both felt the data presented are of interest. However, there was also consensus in the concerns. In the consultation session, the main points of concern focused on:

(1) The novelty of the findings were over-stated, and the manuscript requires a better discussion of work by Maskos et al and Tolu et al showing re-expression of beta2 subunits in VTA rescues nicotine reinforcement in beta2 knock-out mice and that re-expression in both VTA GABAergic and DAergic neurons contribute to nicotine reinforcement. The novelty of the current manuscript is that this is the first demonstration of beta2 sufficiency in nicotine reinforcement in a rat model. The title should also be revised to provide more specific information.

(2) A gain of function needs to be demonstrated in the rat model.

(3) An additional nicotine-naive control is necessary to derive any meaningful conclusions from the voltammetry data.

In addition to the above, the following more specific reviewer points need to be addressed:

Reviewer 1

This manuscript shows that expression of hypersensitive beta 2* nAChRs using a conditional AAV strategy into the VTA of TH-Cre rats results in FR1 self-administration of a low dose of nicotine that does not support acquisition of operant responding in control rats. Following behavioral testing, rats were euthanized and brain slices through nucleus accumbens core were evaluated for nicotine-induced dopamine release.

Strengths:

The hypersensitive nAChR strategy is a good method to show sufficiency of nAChR-mediated activation for nicotine reward-related behaviors in rats.

The study adds to the literature demonstrating a role for beta 2* nAChRs in VTA dopamine neurons in initial rewarding effects of nicotine.

The tools generated will be of use to the field of researchers interested in using molecular genetic approaches to evaluate effects of nAChR subtypes in nicotine-mediated behavioral and physiological effects.

Weaknesses to be addressed:

Major:

1. It is not clear why the authors did not examine nicotine-elicited dopamine release in the voltammetry study. This is an important control to show that expression of the hypersensitive receptor in dopaminergic neurons resulted in increased dopamine dynamics at a low concentration of nicotine.

2. In the voltammetry study, the authors state that they are evaluating, “whether TH-Cre β2Leu9′Ser rats (n=3) with a history of nicotine SA exhibited differential DA dynamics compared to non-Tg rats.” This is a complex experiment in which electrical stimulation in the slice in the presence of hypersensitive nAChRs on DA terminals would elicit both ACh release and depolarization of DA terminals directly. Local ACh release would engage the hypersensitive beta 2 nAChRs on terminals, whereas the authors note themselves that the effect of nicotine self-administration might be to result in plasticity of the DA response itself. Without a control group evaluating the effect of expressing the hypersensitive nAChR expression in a group of rats with no history of nicotine exposure, these two effects cannot be disambiguated. This caveat is mentioned on p. 22, which points to the need for “future studies”, but an additional control experiment here would greatly increase interpretability of this experiment.

3. The difference in number of infusions between rats expressing nAChRs in all VTA neurons (∼90) vs. TH-positive neurons (∼40), the delay in acquisition in the TH-Cre rats until ∼Day 11 and the incomplete extinction once saline is substituted for nicotine is discussed briefly on page 20. Additional discussion should be added here in light of the Tolu et al study in mice showing that rescue of beta 2 expression in DA neurons increases DA release but rescue in both DA and GABA neurons is required for burst firing of DA neurons.

Minor

1. The authors overstate the novelty of the approach and results and should take out statements about, “first to...” throughout the manuscript. These are not necessary and do not represent the work by Maskos et al or Tolu et al accurately. Both those studies (referenced appropriately in the current study) used a replacement strategy in knockout mice to replace the beta 2 subunit in all VTA cellular subtypes or in dopamine and GABA neurons of the VTA, respectively. Those studies showed sufficiency of beta 2* nAChRs in this circuit. The novelty of the current study in using a rat model and the hypersensitive receptor to confirm these findings is more than sufficient for publication.

2. The current study did not use saline control groups and references historical data to identify levels of responding relevant to “acquisition failure.” This is of particular concern for the experiment shown in Figure 2 at the “standard dose” in which response rates are still relatively low in the control groups. The differences between the control and experimental groups at the low dose, as well as the extinction experiment in which saline was substituted for nicotine, greatly mitigate this concern, but a saline control would have been useful.

3. It would have been helpful to see the effects of increasing the ratio from FR1 to FR3 or FR5. Rats will generally achieve more robust levels of responding and the higher FR schedules would ensure that the nicotine self-administration behavior represents active nicotine seeking rather than preventing extinction of food responding. This is a particular issue because of the significant food training used to “accelerate” acquisition.

Reviewer 2

In this manuscript, the authors test the hypothesis that activation of VTA dopaminergic (DAergic) nicotinic acetylcholine receptors (nAChRs) containing the beta2 subunit is sufficient for nicotine reinforcement. Specifically, the authors design cre-dependent viral plasmids to deliver gain-of-function beta2 nicotinic acetylcholine receptors harboring a single point mutation, a serine replacing the 9’ leucine (Leu9’Ser) residue of the pore forming M2 transmembrane domain, that renders nAChRs containing the beta2 subunit hypersensitive to agonist. Plasmids were packaged into AAVs and delivered to the VTA of rats which were then trained to self-administer nicotine intravenously. Interestingly, rats expressing the Leu9’Ser mutated beta2 subunits in the VTA acquired robust self-administration of nicotine at a dose that is too low to support self-administration in control rats. However, at a nicotine dose that does support self-administration in control animals, nicotine intake in Leu9’Ser expressing rats was similar to controls. In addition, selective expression of Leu9’Ser in VTA DAergic neurons in TH::Cre transgenic rats also resulted in robust nicotine self-administration at a low dose compared to controls. Finally, the authors show reduced evoked dopamine release and slower dopamine uptake in slices from Leu9’Ser beta2-expressing TH::Cre compared to control rats after nicotine self-administration. Although it is widely assumed that nAChRs containing the beta2 subunit are both necessary and sufficient for nicotine reinforcement, the data from Leu9’Ser beta2-expressing rats are the first data providing direct evidence that this is true. However, there are controls to consider and points to be clarified. These include:

1. There is no demonstration that Leu9’Ser beta2 expression in VTA results in a gain-of-function nicotinic receptor that is either hypersensitive to agonist and/or resistant to desensitization. Authors could record VTA nicotinic receptor whole-cell currents in response to agonist in midbrain slices from Leu9’Ser beta2-expressing rats compared to control. Alternatively, c-Fos immunohistochemistry could be used to test if low doses of agonist induce c-Fos in the VTA of mutant rats and is blocked by a beta2 nAChR antagonist compared to control. Regardless, it would seem important for the conclusions of the manuscript to show functional data demonstrating at least a shift to the left of the agonist concentration response curve. Caveats to the main conclusion of the manuscript should be discussed (i.e., mutated receptors may be desensitization resistant or result in increased maximal response to agonist so they are not functionally identical to WT receptors except for agonist hypersensitivity. Thus, is activation of beta2 nAChRs really sufficient for nicotine reinforcement?).

2. It is unclear what conclusions can be made from the voltammetry results in figure 4 because not enough groups were examined. Specifically, only rats that self-administered nicotine were compared so whether differences in evoked dopamine release are merely a result of Leu9’Ser beta2 expression cannot be determined (although this is acknowledged in the discussion). In addition, the control used is non-transgenic rats. A more appropriate control may be TH::Cre rats that express cre-dependent eGFP.
